# Altered Resting State Brain Networks in Parkinson’s Disease

**DOI:** 10.1371/journal.pone.0077336

**Published:** 2013-10-28

**Authors:** Martin Göttlich, Thomas F. Münte, Marcus Heldmann, Meike Kasten, Johann Hagenah, Ulrike M. Krämer

**Affiliations:** 1 Department of Neurology, University of Lübeck, Lübeck, Germany; 2 Department of Psychiatry, University of Lübeck, Lübeck, Germany; 3 Department of Neurology, Westküstenklinikum Heide, Heide, Germany; Laureate Institute for Brain Research and The University of Oklahoma, United States of America

## Abstract

Parkinson’s disease (PD) is a neurodegenerative disorder affecting dopaminergic neurons in the substantia nigra leading to dysfunctional cortico-striato-thalamic-cortical loops. In addition to the characteristic motor symptoms, PD patients often show cognitive impairments, affective changes and other non-motor symptoms, suggesting system-wide effects on brain function. Here, we used functional magnetic resonance imaging and graph-theory based analysis methods to investigate altered whole-brain intrinsic functional connectivity in PD patients (n = 37) compared to healthy controls (n = 20). Global network properties indicated less efficient processing in PD. Analysis of brain network modules pointed to increased connectivity within the sensorimotor network, but decreased interaction of the visual network with other brain modules. We found lower connectivity mainly between the cuneus and the ventral caudate, medial orbitofrontal cortex and the temporal lobe. To identify regions of altered connectivity, we mapped the degree of intrinsic functional connectivity both on ROI- and on voxel-level across the brain. Compared to healthy controls, PD patients showed lower connectedness in the medial and middle orbitofrontal cortex. The degree of connectivity was also decreased in the occipital lobe (cuneus and calcarine), but increased in the superior parietal cortex, posterior cingulate gyrus, supramarginal gyrus and supplementary motor area. Our results on global network and module properties indicated that PD manifests as a disconnection syndrome. This was most apparent in the visual network module. The higher connectedness within the sensorimotor module in PD patients may be related to compensation mechanism in order to overcome the functional deficit of the striato-cortical motor loops or to loss of mutual inhibition between brain networks. Abnormal connectivity in the visual network may be related to adaptation and compensation processes as a consequence of altered motor function. Our analysis approach proved sensitive for detecting disease-related localized effects as well as changes in network functions on intermediate and global scale.

## Introduction

Parkinson’s disease (PD) is a neurodegenerative disorder caused mainly by a progressive loss of dopaminergic neurons in the substantia nigra pars compacta projecting to the striatum. The dysfunction of cortico-striatal-thalamic-cortical loops is believed to lead to the hallmark motor features of PD including tremor, akinesia and rigor [1]. Apart from these motor symptoms, a wide range of non-motor deficits can be observed in PD patients. The spectrum of non-motor symptoms in PD includes autonomic dysfunction such as orthostatic symptoms, incontinence, and constipation, hyposmia, REM sleep behavioral disorder and cognitive dysfunction reflecting the multisystem nature of the disorder [2]. Neurocognitive symptoms include working memory deficits, problems in planning and set-shifting as well as affective changes [3], [4], [5], [6].

As PD affects projections to the striatum, it seems obvious to take a network perspective to characterize disease-specific changes in brain functions. Indeed, several studies have analyzed altered connectivity patterns in PD, mainly based on task-related fMRI data [7], [8]. Apart from task-related network changes underlying specific cognitive or motor dysfunctions, the study of resting-state networks allows identifying altered intrinsic BOLD fluctuations as possible disease marker.

A few studies have examined resting-state network changes in PD patients. Helmich et al. [9] assessed functional connectivity within specific cortico-striatal loops based on resting-state data. They found decreased coupling of the posterior putamen and inferior parietal cortex (IPC) in PD patients, but increased coupling between IPC and the anterior putamen. They related this to striatal dopamine depletion in the posterior putamen particularly and took this as evidence for remapping of cortico-striatal loops in PD patients.

Using the graph theoretical measure of a node’s degree, Wu and co-authors [10] studied connectivity changes within the motor network in 22 PD patients. The degree reflects the number of significant functional connections of a node. The authors reported significantly increased or decreased degree values of a number of nodes, which also depended on the current medication status of the patients. Hacker and colleagues [11] investigated resting state functional connectivity of the striatum in Parkinson’s disease. They found markedly lower striatal connectivity with thalamus, midbrain, pons and cerebellum in the PD group compared to healthy controls. In addition they reported a loss of striatal-cortical connectivity to sensorimotor and visual regions in the PD group.

One advantage of graph theory based network analysis is that it provides measures for both global and local connectivity and can thereby be used to assess whole brain networks. A graph can be conceived as a mathematical representation of a network, comprising nodes, i.e. brain regions in our case, and edges, i.e. the connectivity between brain regions [12]. An interesting feature of global connectivity in a number of biological, technological as well as social networks that can be described with graph theory is *small-worldness* [13]. A small-world network is characterized by a low mean shortest path length, comparable to a random network, together with a higher clustering than found in random networks. The path length refers to the number of steps it takes to get from one node to any other node in the network. The clustering coefficient reflects the density of connections between a node’s neighbors [12]. Numerous studies demonstrated that small-world properties are exhibited in brain networks. First observations came from empirical studies on the nervous system of *C. elegans*, cortical networks in the cat and the macaque [14], [15], [16] and then in humans [17], [18]. Recently, small-world properties of brain networks were found using different methods to derive the connectivity between brain regions including functional connectivity [19], [20], cortical thickness [21] and tractography using fiber tracking or diffusion tensor imaging [22], [23].

As many neurological and psychiatric diseases can be considered disconnection syndromes, it is to be expected to find altered brain network metrics as disease markers. Indeed, a number of studies have demonstrated specific changes in global and local connectivity in patients with Alzheimer’s disease (AD), schizophrenia or after focal brain lesions [24], [25], [26]. For instance, AD patients presented reduced global clustering and local clustering in the hippocampi in a resting-state fMRI study [24]. Based on global clustering measures, it was possible to discriminate AD patients from controls [24].

To our knowledge, only one whole-brain resting-state fMRI study in PD patients analyzed graph-based connectivity metrics so far [27]. In their connectivity study based on wavelet correlation, Skidmore and colleagues [27] observed reduced global and nodal efficiency in PD patients, a measure that is inversely related to the mean shortest path length. The nodal efficiency of controls was higher in the supplementary motor cortex, pre-central regions, calcarine cortex and secondary visual areas. This study included only a relatively small number of patients (n = 14), though, and used the brain parcellation scheme of the Automatic Anatomical labeling (AAL) atlas as nodes for their network analysis. This is a quite common approach [24], [27], as it allows parcellating the whole brain including subcortical and cerebellar structures into a still feasible number of regions (n = 116). However, the approach has also been criticized as some of the AAL regions are quite large and heterogeneous and as some known and well-studied networks as the default mode network cannot be replicated with this coarse parcellation [28].

Here, we assessed whole-brain functional connectivity changes in resting-state networks in a large group of PD patients using graph theory metrics. Nodes were derived by further parcellating the AAL regions into modules yielding 343 cortical and subcortical nodes in total (see Zalesky, Fornito et al. [28] for a similar approach). We validate this approach by demonstrating that we can detect the default-mode network using this parcellation method. Moreover, we extend this by calculating the degree as measure of local connectivity also on a voxel-level which provides higher sensitivity to detect small, localized group effects. Whereas previous resting-state fMRI studies in PD mainly used region of interest (ROI) analyses which are very investigator-dependent, we chose a data driven approach. Interestingly, the voxel degree turned out to be a very sensitive marker for altered connectivity. Moreover, the ROI level analysis complements the voxel level analysis in terms of statistical sensitivity, as less statistical comparisons and thereby less rigorous correction for multiple comparisons are needed. We show that brain networks in PD are affected on a larger scale, i.e. scale of brain network modules, and on global scale.

## Materials and Methods

### 1 Ethics Statement

All procedures have been cleared by the ethical committee of the University of Lübeck and all subjects gave their written informed consent prior to participation. The study was performed in agreement with the Declaration of Helsinki.

### 2 Participants

Data was acquired at the University of Lübeck. A total number of 40 PD patients were recruited for the study. In all cases, idiopathic Parkinson’s disease was diagnosed by an experienced neurologist and the severity of clinical symptoms was assessed according to the Unified Parkinson’s Disease Rating Scale (UPDRS). Three patients were excluded due to extensive head motion. The present analysis includes 37 Parkinson’s disease patients (22 male; age: 65±10 years; disease duration 6.5±3.9 years) with advanced disease as indicated by a mean UPDRS score of 22.2±7.7 (part III; clinician-scored motor evaluation). All patients were on medication (L-DOPA, agonists). In total, 21 age-matched control participants were recruited. One control subject was excluded due to extensive head motion. The remaining 20 control subjects (10 male; age: 63±9 years) showed no Parkinsonian signs or any other neurological deficits according to a neurological exam. All subjects included in the study had normal structural images showing no signs of atrophy of the cerebral cortex and subcortical structures. The relevant demographic and clinical information is summarized in Table 1.

**Table 1:** Demographic data on patients and healthy controls.

	Healthy controls	Parkinson’s disease
	n = 20	n = 37
Gender men/women	10/10	22/15
Age in years (s.d.)	63 (9)	65 (10)
UPDRS part III (motor)	n/a	22.3 (7.7)
Disease duration (yr)	n/a	6.5 (3.9)

Notes: UPDRS = Unified Parkinson’s Disease Rating Scale.

### 3 Experimental Design

The functional MRI data was acquired during a so-called resting-state block. Subjects were instructed to neither engage in any particular cognitive nor motor activity and to keep their eyes closed. The functional run took 6 minutes to complete.

### 4 Image Acquisition

Structural and functional MRI images were recorded on a Philips Achieva 1.5-T scanner (Philips Healthcare, the Netherlands). A total of N = 178 functional images were acquired using a single-shot gradient-echo echo-planar imaging (EPI) sequence sensitive to blood oxygen level dependent (BOLD) contrast (volume TR = 2000 ms, TE = 50 ms, spatial resolution 3×3×4 mm^3^, 2 mm interslice gap, image matrix 64×64×21, standard 8-channel head coil). High resolution structural images were obtained applying a T1-weighted 3D turbo gradient echo sequence with SENSE (image matrix 256×233×170, 1 mm isovoxel).

### 5 Preprocessing

Preprocessing was performed using the SPM8 software package (http://www.fil.ion.ucl.ac.uk/spm/). The first 10 images of each dataset were discarded to allow for magnetization equilibrium and for the subjects to adjust to the environment.

The preprocessing included the following steps: (i) Correction for differences in the image acquisition time between slices; (ii) a six parameter rigid body spatial transformation to correct for head motion during data acquisition; (iii) co-registration of the structural image to the mean functional image; (iv) grey and white matter segmentation, bias correction and spatial normalization of the structural image to a standard template (Montreal Neurological Institute); (v) In order to reduce the influence of motion and unspecific physiological effects, a regression of nuisance variables from the data was performed. Nuisance variables included white matter and ventricular signals and the six motion parameters determined in the realignment procedure. (vi) spatial normalization of the functional images using the normalization parameters estimated in the previous preprocessing step and resampling to 2 mm×2 mm×2 mm; (vii) spatial smoothing with a 6 mm full width half maximum Gaussian kernel. (viii) A temporal bandpass filter was applied to all voxel time series (0.01 Hz<f <0.08 Hz).

Subjects with strong head motion were excluded from the analysis. The six realignment parameters, i.e. three displacements and three elementary rotations with respect to the first image in the EPI series, were used as an estimator for the head motion. The displacements were required to be smaller than 3.0 mm (minimum to maximum) and the individual rotations smaller than 3.0 degrees. Subject showing any displacement or rotation greater than these cut-offs was excluded. Instantaneous motion has a confounding effect on the measurement of functional connectivity [29], [30]. As an indicator for instantaneous motion, we used the framewise displacement as described by Power and colleagues (2012):
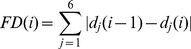
d_j_(i) are the six motion parameters determined for each scan where i indexes the frame. The rotation parameters were converted from degrees into millimeters by calculating the evoked displacement on the surface of a sphere with a radius of 50 mm. The chosen radius of 50 mm corresponds in good approximation to the mean distance of the cerebral cortex to the center of the head. Subjects with a framewise displacement of more than 2.5 mm were excluded from the analysis since instantaneous motion of this magnitude can affect the connectivity matrix considerably. Three patients and one control were excluded because of these requirements, leaving 37 patients and 20 controls for the analyses.

### 6 Voxel Degree Maps

Voxel degree maps were calculated by correlating the temporal BOLD signal fluctuation of each voxel with all other voxels in the brain and counting the number of connections above a certain threshold. As a measure for the temporal correlation, we computed the zero-lag Pearson’s linear correlation coefficient *r*. The individual correlation coefficients were entered into an N×N adjacency matrix where N is the number of voxels. The voxel network matrix was thresholded by 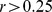 suppressing random correlations. This results in a binary undirected network matrix 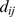. The voxel degree 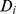 was derived from the network matrix as follows:
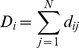



The degree maps were z-transformed to allow for averaging and between subject comparisons:
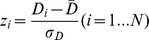



Here, 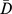 denotes the mean degree and 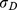 the standard deviation. No distinction is made between local and long-range connectedness. Brain regions showing high z-scores are interpreted as hub regions, i.e. regions which are highly connected and thus play a key role for the network integration. In a previous study the posterior cingulate, lateral temporal, lateral parietal, and medial/lateral prefrontal cortex were identified as prominent hubs [31]. These are regions related to the default mode network.

### 7 Graph Formation

A graph consists of a set of nodes which are connected by edges. In this section we introduce our approach to define the nodes and edges describing the brain network. Nodes represent brain regions, i.e. a collection of voxels which are spatially and functionally connected.

#### 7.1 Nodes

A regional parcellation of each hemisphere of the brain into 45 regions was performed according to the Automatic Anatomical labeling template as described by Tzourio-Mazoyer and colleagues [32]. This template is based on an anatomical parcellation according to major sulci and gyri using a spatially normalized single subject high resolution T1 volume provided by the Montreal Neurological Institute (MNI) [33]. The AAL regions (or AAL regions of interest, AAL ROIs) were further parcellated yielding 343 cortical and subcortical nodes in total (Figure S1). For each AAL region and each subject we obtained a connectivity matrix by correlating the temporal voxel BOLD signal fluctuations. As a measure for the connectivity between the voxels we used the Pearson correlation coefficient. The Pearson correlation coefficients were Fisher z-transformed and the mean correlation matrix was calculated by averaging all connectivity matrices within the control group. The connectivity matrix was thresholded in order to suppress spurious correlations keeping 30% of strongest weights (proportional threshold of S = 0.3). Structures within the AAL regions were identified using Newman’s spectral algorithm [34] applied to the mean correlation matrices. This algorithm subdivides a network matrix into non-overlapping groups of nodes, i.e. modules, in a way that maximizes the number of edges falling within modules minus the expected number in an equivalent network with edges placed at random.

These modules served as nodes for the brain network analysis. In the following we refer to the new regions as sub-AAL regions.

#### 7.2 Edges

The functional connectivity between brain regions was established by correlating the regional mean time courses. As a measure for the temporal correlation, we computed the zero-lag Pearson’s linear correlation coefficient. The correlation coefficients were inverse hyperbolic tangent transformed (Fisher z-transformed) and entered into the correlation matrix as basis for the network analysis.

### 8 Graph Analysis

A graph theoretical approach was used to investigate both local and global properties of the brain network. A network can be represented by a set of nodes, here brain regions, which are connected by edges. Edges reflect interactions between brain regions which are defined in this case by the correlation as described above. The symmetric correlation matrices represent undirected weighted graphs. In this work we used binary in contrast to weighted graphs. The conversion from a weighted graph to a binary graph was performed by thresholding at a specific sparsity value with sparsity reflecting the number of edges relative to all possible edges. The choice of the sparsity threshold has a major effect on the network topology. Instead of arbitrarily choosing one single sparsity value, we present network metrics which are based on binary graphs as a function of the sparsity *S*. We considered sparsity values between S_min_ = 0.1 and S_max_ = 0.35. The lower limit is given by the requirement that all graphs are connected, i.e. there are no nodes which are isolated from the rest of the network. The upper limit was chosen such that the contribution of spurious correlations is strongly suppressed and the resulting graphs exhibit small-world properties. For most graph theoretic quantities there are alternative algorithms which work on weighted adjacency matrices. We decided to use binary versions of the algorithms consistently throughout the study. One reason being their simplicity and the other being that also weighted matrices have to be thresholded to remove negative weights and to suppress spurious correlations. This threshold is also to some degree arbitrary and has to be justified and varied to show that the results do not depend on the choice of the threshold.

#### 8.1 Degree, clustering coefficient and characteristic path length

Once a graph is created, numerous measures describing its topological properties can be computed [35]. The node degree *D_i_* is defined as the number of connections to other nodes in the network given a sparsity threshold S. Network hubs are usually characterized by a high degree in comparison to other nodes in the network. The node degree is z-transformed according to the following formula:
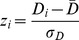




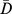
 and 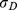 are the mean and the standard deviation of the degree distribution, respectively.

The clustering coefficient characterizes the brain network on a local level, whereas the mean clustering coefficient and the characteristic path length are used to characterize the brain network on a global level. The clustering coefficient *C_i_* of a node *i* is a measure of the local network connectivity. It is defined as the fraction of a node’s neighbors which are neighbors of each other. We compute the clustering coefficient by applying an algorithm which operates on binary graphs [13]. The characteristic path length *L* is defined as the average shortest path length in the network, where the path length *d_ij_* between two nodes *i* and *j* is given by the number of nodes which have to be passed to transfer information from node *i* to node *j*. While the mean clustering coefficient is a measure for the segregation of the network, the characteristic path length is a measure for the global integration and thus the efficiency of the network.

#### 8.2 Network topology

The mean clustering coefficient and the characteristic path length indicate whether the nodes of a complex network are connected in a random or small-world order. A small-world network in comparison to a random network is characterized by a considerably higher mean clustering 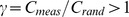 and a comparably short characteristic path length 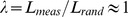 [13]. Therefore, small-world networks are both globally and locally efficient in transferring information [36]. In order to calculate the network metrics γ and λ, we constructed random reference networks applying an algorithm which randomly rewires the measured networks while preserving the degree distribution [37].

#### 8.3 Community structure and participation

Community structures can be considered an intermediate level of network organization. A graph community structure is a subdivision of a network into non-overlapping groups of nodes (modules) which are densely connected with each other but less so with other nodes in the network [12]. Here, we used Newman’s spectral algorithm to identify brain network modules [34]. Given a previously determined community structure, the participation coefficient is a measure of the diversity of inter-community connections of individual nodes. A node with a high participation coefficient is strongly connected to nodes in other communities working as a hub which connects different communities. The modular structure of the brain network was derived from the control group by averaging the individual network matrices. The mean network matrix was then thresholded at different sparsity values and a community structure was identified applying Newman’s spectral algorithm [34]. The community structure was applied to the network matrices on an individual subject level and the groups’ mean clustering coefficient and participation coefficient were calculated for each module. In addition we investigated group differences in the participation coefficient for individual nodes.

The network metrics and the reference networks were calculated using the Brain Connectivity Toolbox (BCT, http://www.brain-connectivity-toolbox.net/). The networks were visualized with the BrainNet Viewer (http://www.nitrc.org/projects/bnv/).

### 9 Statistical Analysis

Differences in the voxel degree between healthy controls and PD patients were investigated by a random effects analysis applying a two-sample t-test. Statistic images were assessed for cluster-wise significance using a cluster-defining threshold of p = 0.005; the 0.05 FDR-corrected critical cluster size was k = 184 [38], [39]. The analysis was performed using SPM8 (http://www.fil.ion.ucl.ac.uk/spm/).

Statistical tests for significant between-group differences in graph theoretical quantities, i.e. degree, clustering coefficient and characteristic path length, were carried out by nonparametric permutation tests [40]. When testing for group effects in node properties, 0.05 FDR-corrected results are presented [41], [42].

## Results

The main focus of our study was on altered whole brain network organization in PD. As we chose a new parcellation approach for our study, we first investigated the validity of our parcellation approach by contrasting results for voxel and sub-AAL ROI level analyses. We then compared patients and controls regarding global network measures and investigated changes in brain network organization at an intermediate scale, i.e. the level of network modules. We also examined the relation between observed connectivity changes on module level and patients’ clinical status (UPDRS; part III). To validate group differences in network modules, we additionally examined group differences in node degree on voxel-level. Finally, to exclude spurious group differences, we thoroughly investigated the influence of head motion.

### 1 Validation of Methodological Approach

**Figure 1:**
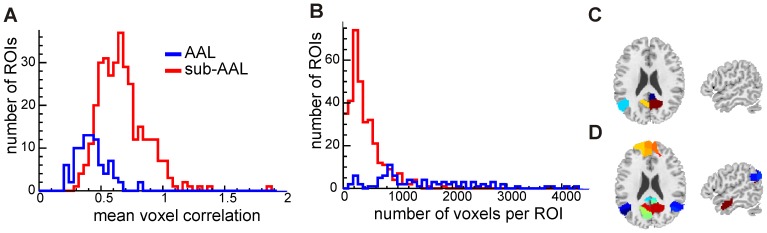
Comparison of the two parcellation approaches. A) The mean voxel correlation (Fisher transformed) within the ROIs for the parcellation according to the AAL atlas and the parcellation derived from the modular structure within the AAL regions (sub-AAL). B) The number of voxels per ROI for AAL and sub-AAL parcellation is plotted. C) ROIs significantly correlated to the left posterior cingulate cortex (PCC) ROI for the AAL parcellation. D) ROIs significantly correlated to the seed ROI in the left PCC for the sub-AAL ROIs.

The average number of modules identified in the AAL regions was four (range 3 to 5). Figure 1A shows the mean voxel correlation (ROI homogeneity) within each region for the AAL parcellation compared to the new approach. Correlation coefficients are Fisher transformed. We observed a higher ROI homogeneity for the sub-AAL regions. The mean ROI homogeneity for the AAL parcellation was 0.42 compared to 0.67 for the sub-AAL regions. In Figure 1B we depict the number of voxels within each region. The sub-AAL regions are less heterogeneous in terms of size than the AAL regions. These improvements are reflected in the connectivity patterns derived from the brain network matrices as demonstrated in Figures 1C and 1D. Shown are the regions connected to the left posterior cingulate cortex (proportional threshold of S = 0.1) for the AAL and the sub-AAL parcellation, respectively. Only for the new parcellation approach, we obtain a connectivity pattern which includes the complete default mode network including posterior cingulate, lateral temporal, lateral parietal and medial prefrontal regions.

**Figure 2:**
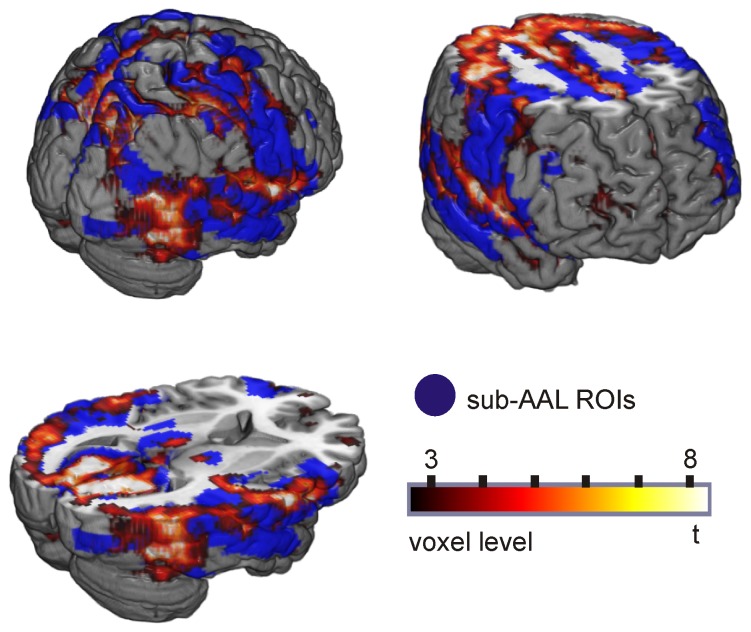
Brain regions with high degree centrality in controls. Shown are the results from voxel-level (red to white scale) and ROI-level (blue) analyses.

We identified hub regions in controls based on voxel level analyses and sub-AAL ROI level analyses to qualitatively assess the comparability of these two approaches. Figure 2 shows an overlap of voxels and sub-AAL regions with a high z-degree (one-sample t-test; 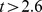). A very good agreement between the two approaches can be observed, i.e. similar hub regions are identified: lateral frontal, parietal, lateral temporal cortex and thalamus. Table S1 summarizes the results on voxel-level. Whereas hubs identified on the ROI level span generally larger regions, smaller hub regions in, for instance, lateral prefrontal cortex are detected using voxel level analyses only. Our results are in accord with findings of hub regions reported by other groups [19], [31].

### 2 Global Network Properties

**Figure 3:**
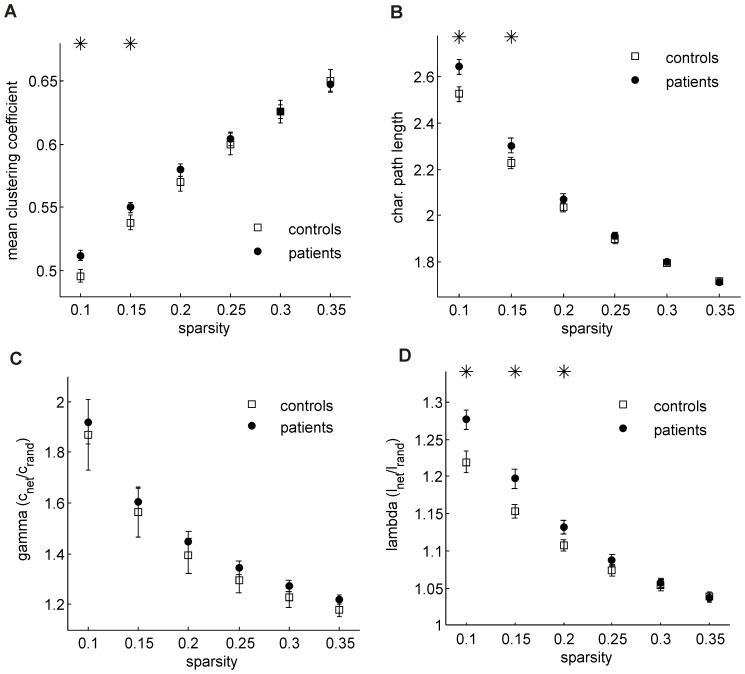
Global network properties for Parkinson’s disease patients (PD, full circles) and healthy controls (squares) as a function of the sparsity. A) mean clustering coefficient B) characteristic path length C) clustering coefficient normalized to a reference random network D) characteristic path length normalized to a reference random network. The asterisks indicate significant between-group differences (permutation test, α = 0.05).

Figures 3A and 3B show the mean clustering coefficient and the characteristic path length as a function of the sparsity for the control and the patient group. The network metrics are calculated for each of the individual functional connectivity graphs. Shown are the groups’ mean values and the standard errors. A nonparametric permutation test was applied to test the statistical significance of between-group differences (α = 0.05). A significantly higher clustering and characteristic path length is observed in the patient group for low sparsity values (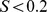). The increased characteristic path length indicates a less efficient organization of the brain network for patients suffering from Parkinson’s disease. Figures 3C and 3D depict the normalized clustering coefficient γ and characteristic path length λ, respectively. The higher clustering and the similar characteristic path length compared to reference random networks suggest a small-world organization of the functional brain networks both in controls and in PD patients.

### 3 Community Structure and Participation

**Figure 4:**
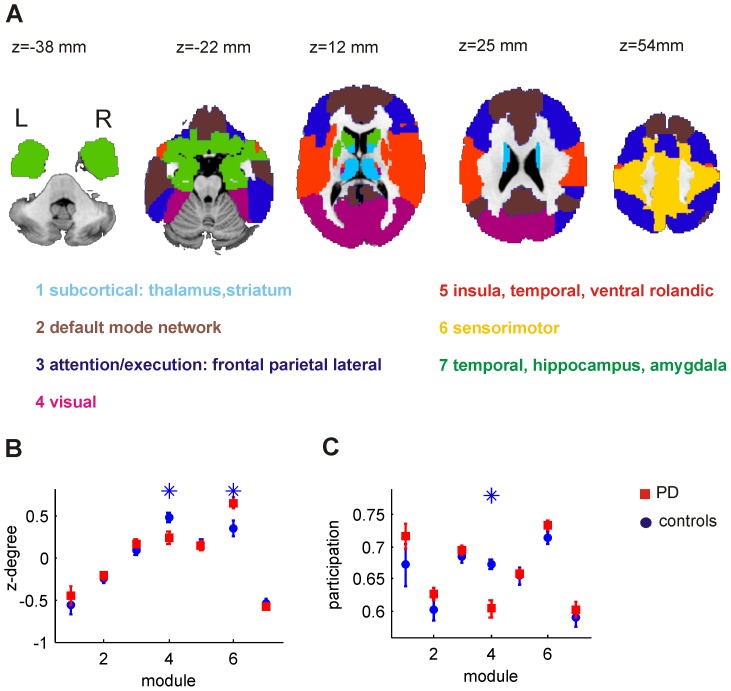
Modular structure of the brain network. A) Overview of the different network modules. B) mean z-degree of nodes comprising module C) mean participation nodes comprising module for patients (red squares) and controls (blue circles).

In addition to the global differences in path length and clustering, we investigated differences on the intermediate level of brain network organization by comparing properties of ROI communities. The community structure of the brain network was derived from the mean network matrix obtained from the control group by averaging the connectivity for each edge. A proportional threshold of S = 0.2 was applied to the mean correlation matrix. This analysis yielded seven communities as depicted in Figure 4A. The individual modules can be related to known resting state networks (default mode, visual, sensorimotor, attention, sub-cortical) [43]. The default mode (module 2; brown) and visual (module 4; purple) networks were clearly identified. We also observed communities related mainly to the sensorimotor network (module 6, yellow) and executive/attention networks (modules 3 and 5; blue and orange). Module 1 (light blue) comprised subcortical structures only (thalamus and striatum ROIs). For each module we investigated group differences in the mean degree and participation. We found a significantly higher degree in the visual network for controls compared to PD patients (Figure 4B). This can be explained mainly by stronger long-range connectivity to other modules as indicated by a significantly higher participation coefficient of controls observed in that module (Figure 4C). By contrast, the sensorimotor network exhibits a significantly larger degree for the PD group. This is mainly a consequence of stronger local connections within the module itself since we observe no difference in the participation for module 6 (Figure 4C). It should be stressed that the observed between-group effects were independent of the choice of the sparsity bin when applying the same modular structure to the brain network.

### 4 Group Differences in Node Degree and Edges

**Figure 5:**
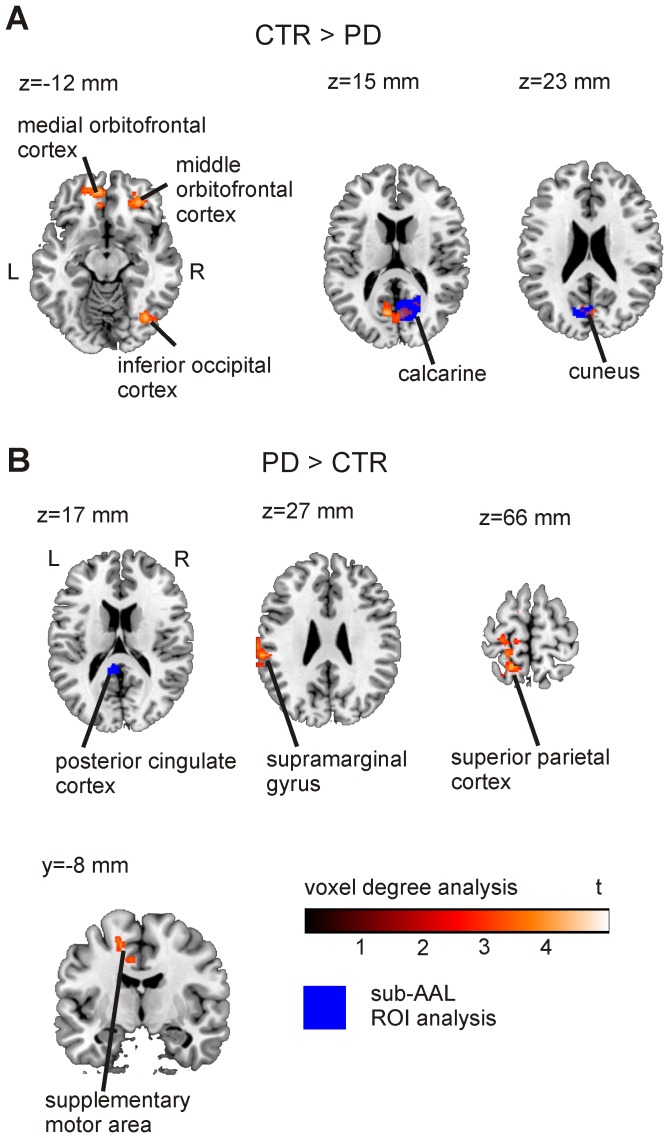
Between group effects in z-degree. Voxel-level (assessed for cluster-wise significance using a cluster defining threshold of p = 0.005; 0.05 FDR-corrected) and ROI-level data (blue areas; 0.05 FDR corrected; sparsity S = 0.2) are presented. A) Regions with a larger degree in healthy controls compared to patients. B) Regions with a larger degree for PD patients compared to controls.

We tested each node (sub-AAL ROI) for significant between-group effects in z-degree (0.05 FDR-corrected). Two nodes, one in the calcarine and one in the cuneus, showed a significantly lower degree for PD patients. One node in the posterior cingulate cortex exhibited a higher degree for PD patients with respect to healthy controls. The nodes are depicted in Figure 5 and the results are summarized in Table 2 (sparsity S = 0.2), where we listed ROI center of mass coordinates, mean degree and adjusted p-values. It should be stressed that these findings are independent of the choice of the sparsity as shown in Figure S2 where we investigated the mean degree for PD patients and healthy controls as a function of the sparsity.

**Table 2:** Nodes showing the strongest between group effects in degree centrality.

Anatomical region	ROI center			degree		p-value
	(x y z) [mm]			CTR	PD	(adjusted)
**CTR > PD**						
Calcarine R 3	14	−62	12	1.4	0.6	0.022
Cuneus L 1	−7	−76	29	1.3	0.7	0.042
**PD > CTR**						
Cingulate Cortex Post L 3	−8	−43	15	−1.0	−0.2	0.042

**Notes:** Network nodes showing the strongest between group effects (permutation test; α = 0.05 FDR-corrected). The anatomical region, the center of mass MNI coordinates of the ROIs, the node degree for controls (CTR) and patients (PD) and the adjusted p-values are listed.

The modular structure of the brain networks and observed group differences in modules 4 and 6 rely on the parcellation in ROIs. To validate this approach and verify group differences in node degree, we additionally ran a random effects analysis to investigate group differences in the voxel-degree. Statistics t-images were assessed for cluster-wise significance using a cluster-defining threshold of p = 0.005; the 0.05 FDR-corrected critical cluster size was 184. The results are depicted in Figure 5 and summarized in Table 3 which lists the cluster size, cluster-level p-value (0.05 FDR-corrected), peak t-value as well as the peak location in MNI coordinates. In Figure 5, group differences in voxel-level degree maps are shown together with ROI-level results to allow direct comparison of the two approaches. On voxel level, we observed an increased z-degree in controls relative to patients in the occipital cortex. In addition, voxel-level analyses showed reduced degree scores in patients in medial orbitofrontal areas, important target regions of dopaminergic projections [44], [45], [46], [47], [48]. By contrast, a higher degree for PD patients relative to controls was found in the pre-central, post-central and parietal cortex as well as in the supplementary motor area.

**Table 3:** Between-group differences in voxel degree.

Anatomical region	p (adj.)	k	local maxima			T
	(cluster)		(x y z) [mm]			(peak)
***CTR > PD***						
Calcarine; Cuneus R	0.000	1015	22	−60	12	4.63
Frontal Inf Orb R	0.006	321	30	38	−10	4.35
Frontal Med Orb L	0.007	293	−8	48	−10	4.19
Occipital Inf R	0.047	184	34	−72	−8	4.14
***PD > CTR***						
Parietal Sup L	0.000	738	−18	−52	62	4.94
SupraMarginal L	0.045	212	−66	−22	34	4.93
Supp Motor Area L	0.005	346	−12	−8	52	4.18

**Notes:** Clusters where differences in the voxel degree between patients and controls are observed (cluster defining threshold p<0.005; the 0.05 FDR-corrected critical cluster size was 184). Anatomical region, cluster level probability (0.05 FDR-corrected), number of voxels per cluster (k), local maxima in MNI coordinates and peak T-scores are listed.

To allow for better comparability between the voxel- and ROI-based analyses we also report uncorrected results on both voxel- and ROI-level in the supplementary materials (Figure S3; Tables S2 and S3). We find a satisfactory agreement between the two approaches. Note, that in Figure S3 we only tested ROIs comprising the modules 4 and 6.

Note, that the absolute threshold of r = 0.25 applied in the voxel-level analysis is not equivalent to the proportional threshold of S = 0.2 in the ROI-analysis. We showed that our results did not depend on the choice of the threshold (Figure S2). It is thus valid to compare the results of the two approaches qualitatively.

**Figure 6:**
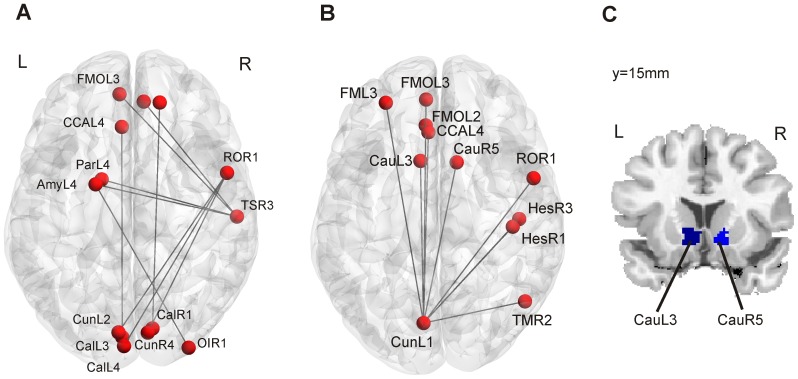
Altered connectivity of the visual network. A) Altered connectivity of nodes in module 4 (visual network). We observe stronger occipital-frontal and occipital-temporal connectivity for healthy controls. B) Altered connectivity of the cuneus node CunL1. C) Coronal view showing the anatomical location of the two caudate nodes CauL3 and CauR5. Abbreviations: Amy: Amygdala; Cal: Calcarine; Cau: Caudate; Cun: Cuneus; CCA: anterior cingulate cortex; FMO: medial orbitofrontal cortex; Hes: Heschl gyrus; OI: inferior occipital cortex; Par: parahippocampus; RO: rolandic operculum; TM: medial temporal gyrus; TS: superior temporal gyrus.

Controls showed a significantly higher degree in module 4 (visual network) and a significantly higher participation coefficient in this module, which indicated a stronger connectivity to nodes in other modules. To further examine which connections are contributing to this finding, we show the ten edges from nodes in the visual module to other nodes in the network which differed most between groups (established through one-tailed t-test). We found significantly stronger connectivity of the visual network to subcortical, temporal and medial frontal brain regions (Figure 6A; Table 4). We then applied the same strategy to better understand the altered connectivity of the calcarine and cuneus nodes where we observed significant between group effects in the degree. Figure 6B (Table 5) shows the ten edges where we observed the strongest effects. None of these connections involved the calcarine. We found stronger connectivity of the cuneus node to medial orbitofrontal and to ventral caudate nodes. The anatomical location of the two caudate nodes is depicted in Figure 6C. In Tables 4 and 5 we summarized the results and listed the mean connectivity for each group and the corresponding p-values according to a two-sample t-test.

**Table 4:** Altered connectivity of nodes within module 4 to nodes outside module 4.

ROI 1	ROI 2	CTR	PD	p
Calcarine L 4	Cingulate CortexAnt L 4	0.28	−0.01	0.00002
Calcarine R 1	Frontal Sup Orb R 4	0.33	0.07	0.00003
Amygdala L 3	Occipital Inf R 1	0.51	0.14	0.00001
Cuneus L 2	Rolandic Oper R 1	0.45	0.14	0.00001
Cuneus L 3	Rolandic Oper R 1	0.31	0.01	0.00006
Cuneus R 4	Rolandic Oper R 1	0.44	0.15	0.00002
Amygdala L 3	Temporal Sup R 3	0.53	0.14	0.00020
Frontal Med Orb L 3	Temporal Sup R 3	0.53	0.15	0.00001
Frontal Med Orb R 2	Temporal Sup R 3	0.48	0.12	0.00001
ParaHippocampal L 4	Temporal Sup R 3	0.42	0.15	0.00010

**Notes:** Listed are the two nodes defining an edge. Mean connectivity in control group (CTR) and patients (PD) and corresponding p-values are listed.

**Table 5:** Altered connectivity of left cuneus node with significant between-group effects.

ROI 1	ROI 2	CTR	PD	p
Caudate L 3	Cuneus L 1	0.44	0.23	0.0001
Caudate R 5	Cuneus L 1	0.32	0.13	0.0005
Cingulate CortexAnt L 4	Cuneus L 1	0.34	0.13	0.0019
Cuneus L 1	Frontal Med Orb L 2	0.37	0.13	0.0003
Cuneus L 1	Frontal Med Orb L 3	0.49	0.25	0.0011
Cuneus L 1	Frontal Mid L 3	0.55	0.34	0.0017
Cuneus L 1	Heschl R 1	0.54	0.27	0.0016
Cuneus L 1	Heschl R 3	0.46	0.21	0.0005
Cuneus L 1	Rolandic Oper R 1	0.43	0.20	0.0003
Cuneus L 1	Temporal Mid R 2	0.91	0.66	0.0006

**Notes:** Listed are the two nodes defining an edge. Mean connectivity in control group (CTR) and patients (PD) and corresponding p-values are listed.

The stronger connectivity of occipital to medial frontal brain regions might contribute to the higher voxel degree in medial frontal cortex as depicted in Figure 5.

### 5 Influence of Head Motion

Head motion influences the measurement of the intrinsic functional connectivity. Several recent studies report decreased long range connectivity and increased local connectivity due to motion [29], [30]. Since we observed weakened long-range connectivity in case of the PD group, we studied extensively if our results were confounded by head motion. In order to verify that the observed group differences were not caused by differences in motion, we tested whether the extent of motion was correlated with the functional connectivity between brain regions. We used four different parameters to quantify the extent of motion:

1. The maximum head displacement *D_max_* derived from the six parameters of the rigid body transformation during the realignment procedure *d_j_*. The parameters describing the rotations are multiplied by R = 50 mm.
2. The root mean square of the head motion *D_RMS_*:

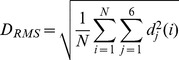



Here, i indexes the N frames and is the displacement of frame i for the realignment parameter j.

1. The maximum framewise displacement *FD_max_*.
2. The root mean square of the framewise displacement *FD_RMS_*:

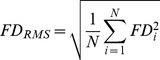



In Table 6 the group mean and standard deviations are given for the four different parameters quantifying the extent of head motion. According to a one-tailed two-sample t-test no significantly stronger head motion was found in the PD group (α = 0.05), although a trend was found in three of the parameters.

**Table 6:** Head motion parameters.

Quantity	CTR	PD	p-value	Significant
Max motion [mm]	0.81 (0.61)	1.08 (0.56)	0.06	2 (1 negative)
RMS motion [mm]	0.60 (0.55)	0.82 (0.60)	0.09	0 (0 negative)
Max FD [mm]	0.88 (0.59)	1.13 (0.61)	0.07	1 (0 negative)
RMS FD [mm]	0.28 (0.15)	0.28 (0.15)	0.47	1 (0 negative)

**Notes:** Maximal motion, root mean square (RMS) motion, maximal framewise displacement (FD) and root-mean square framewise displacement (RMS FD) for controls (CTR) and patients (PD). Quoted are the mean values and their standard deviations. We performed a two-sample t-test to test for between-group effects. The p-values are quoted. We also listed the number of edges which significantly correlated to each of the motion parameters.

Any effect in graph properties found in this study ultimately depends on the validity of the network matrices. In order to test if differences in the network matrices between controls and PD patients are confounded by head motion we tested if the connectivity strength is correlated to any of the four parameters used to quantify the extent of head motion. First, we selected edges showing a significant group effect according to a two-sample t-test and found 370 edges at a significance level of α = 0.001. In a second step, we correlated each of the four head motion parameters to the connectivity strength of the selected edges. Table 6 lists the number of significant correlations (α = 0.01; four expected under null-hypothesis). Only one negative correlation was observed between maximum motion and connectivity between cuneus and medial orbitofrontal cortex. This is well compatible with the number of false discoveries expected under the null-hypothesis. None of the other connectivity values correlated with motion parameters, which is evidence that measured functional connectivity differences cannot be explained by head motion. Critically, we did not find any hint for long-range connections to be negatively correlated to one of the four measures of head motion.

Furthermore, the z-degree in the nodes where we found significant group effects (Table 2) did not correlate to any of the four parameters used to quantify the extent of head motion.

## Discussion

We investigated whole-brain resting-state network changes in PD patients using several graph-theory based measures. First, we investigated global network properties as the characteristic path length and the mean clustering coefficient, which indicated less efficient brain network topology in PD. Changes in the brain network organization on an intermediate scale were quantified by examining communities of ROIs derived from the modular structure of the brain network. PD patients exhibited a significantly lower module degree in the visual network (module 4; Beckmann et al [43]) and a significantly higher degree in a sensorimotor network (module 6; [43]). The visual network module also showed a reduced participation coefficient in PD patients, resulting from reduced frontal-occipital and frontal-subcortical (ventral caudate) connectivity. We were able to validate these results with an alternative methodological approach by studying the node degree on voxel level. PD patients showed decreased connectivity in orbitofrontal and occipital regions as well as in the caudate and increased connectivity of sensorimotor and parietal brain regions. The voxel level analyses controlled for potential analysis biases introduced by choosing large ROIs as network nodes. The good agreement between the two analysis approaches strengthens the confidence in the results of the network analysis. In the following, we first discuss methodological issues pertaining to our parcellation approach and to the influence of head motion, before we discuss the main results of altered brain networks in PD.

### 1 Validation of the Parcellation Approach

We used the brain regions defined in the anatomical labeling template [32] as a starting point for our whole-brain parcellation. The AAL regions were subdivided according to their community structure yielding 343 regions. This parcellation approach resulted in an increased ROI homogeneity as well as a more similar ROI size when compared to the AAL template (Figure 1). Previous resting-state fMRI brain network analyses were mainly based on a parcellation according to the AAL template as reviewed by Zalesky and colleagues [28]. Zalesky et al. [28] investigated how various network organizational parameters depend on the spatial scale of the brain parcellation. In contrast to our approach they applied a random parcellation of AAL regions. We argue that our approach of clustering voxels that are correlated leads to higher functional homogeneity at a given spatial scale.

We validated our approach by showing that the default-mode network can be replicated much better compared to a parcellation according to AAL ROIs. Furthermore, the typical resting-state networks that have been identified previously by independent component analyses [43], [49] were revealed through the analysis of the community structure of the group mean network matrix. Another independent validation was performed by comparing the voxel-based degree map to the ROI-based degree map. We found a very good agreement between the two approaches. This justifies using this parcellation approach to study the connectivity between brain regions and to investigate more complex network properties which are computationally extensive, e.g. the characteristic path length or the clustering coefficient. Furthermore, the problem of multiple testing is enormously reduced due the much smaller number of network nodes.

On the other hand, we expect losing sensitivity for effects in regions which are small compared to the ROI size and for effects in regions outside the AAL template such as the midbrain. This was also observable in the present study in which small, localized group differences such as in caudate head and orbitofrontal cortex were detectable in voxel level analyses only. To examine brain network changes in neurological and psychiatric disorders, we therefore suggest using a combined analysis scheme employing voxel-based and sub-AAL ROI-based analyses to capture both small, localized effects and more complex network properties on an intermediate and global network level.

### 2 Effects of Head Motion

As PD is a movement disorder and connectivity measures have been shown to be particularly susceptible to movement artifacts, we thoroughly investigated the influence of head motion on the measurement of functional connectivity. Results indicated that the connectivity measures did not depend on any of four different measures of head motion. We also found no indication for an effect of head motion on the degree in any of the nodes where we found significant between-group effects. Moreover, Power and colleagues [30] quantified the changes in the correlation coefficients between different brain regions which can be explained by motion and gave a conservative upper limit of about 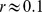. The large differences in functional connectivity (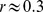) found in this study are thus not likely to be caused by motion. Furthermore, we observed mainly altered connectivity between frontal-occipital and subcortical-occipital regions but no differences in the connectivity between left and right temporal regions. We neither observed short-range connections to be consistently stronger for the PD patients, which would be expected if the connectivity measurement was affected by head motion. If the measurement of the functional connectivity was biased by head motion we would expect to find an altered node degree, node participation and a higher local clustering in all brain regions but in contrast we find effects to be localized in certain brain regions (Figure 5). Together, this speaks against differences in head motion as reason for group differences in network measures.

### 3 Network Topology

Global network measures, i.e. the normalized mean clustering coefficient and characteristic path length, indicated a small-world organization of the brain network in controls as well as in PD patients. However, the larger characteristic path length observed in the PD group compared to controls points to an altered organization of the brain network leading to lower network efficiency. We were thus able to verify the observation of lower network efficiency for PD patients as reported by Skidmore and colleagues [27]. Note that the characteristic path length is inversely related to the global efficiency [35] allowing for a direct comparison of the two studies concerning the network efficiency. Similar observations have also been made in studies on other neurological diseases, e.g. Alzheimer’s disease [50]. The analysis of network modules allowed us to obtain deeper insights into the global network changes as will be discussed in the following.

To investigate altered brain network organization in PD on an intermediate scale of network organization we explored properties of ROI communities. The communities which we identified in the data resembled to a large extent well-known resting state networks or combinations of them (RSN) [43]. The manifestation of PD as a disconnection syndrome became apparent in the visual RSN where we observed a significantly higher degree and network participation for healthy controls in comparison to the PD group. It should be stressed that in the context of this study we refer to functional disconnection in contrast to structural disconnection. Visual deficits in Parkinson’s disease [51], [52] range from problems in basic perceptual and semantic visual processing at an early stage of cognitive deterioration [53], deficits in orientation and motion discrimination [54], [55] to visual hallucinations [56], [57]. We speculate that our finding of a reduced functional connection of the visual network to subcortical, temporal and frontal areas is related to visual deficits frequently reported in PD [51]. In the case of altered motor function, the visual system also needs to adapt and compensate which might additionally contribute to altered network connectivity. Targeted studies are necessary to shed light on the relation of our observations to specific visual deficits or compensatory mechanisms.

We interpret our finding of a higher degree within the module related to the sensorimotor RSN (module 6) as a compensation mechanism in order to overcome the functional deficit of the striato-cortical motor loops [58], [59], [60], [61]. This is in line with higher local connectivity in sensorimotor and premotor areas reported by Wu et al. [62] investigating the regional homogeneity in PD. Similarly, Sabatini et al. [63] reported a cortical motor reorganization in PD expressed by a hyperactivation in the posterior SMA, the anterior cingulate cortex and the primary sensorimotor cortices during a complex sequential motor task.

### 4 Local Network Changes in PD

Regions with high degree centrality are believed to act as hubs integrating the functionality of different brain regions in healthy individuals. We identified the following hub regions: lateral frontal, parietal, lateral temporal cortex and thalamus. These results are in agreement with findings reported by other groups [19], .

When comparing healthy controls to the PD group on voxel level, we found a lower connectivity for patients in brain regions involved in dopaminergic pathways, i.e. caudate head, hippocampus and the orbitofrontal cortex [44], [45], [46], [47], [48], [64] but also in the occipital lobe. Group differences of the degree in the calcarine and the cuneus were seen both in voxel-based and ROI-based analyses. The agreement between voxel- and ROI-based analyses became more evident when we applied less stringent p-value thresholds. PD-related differences in connectivity degree were associated with weaker network edges between orbitofrontal and occipital cortex as well as between subcortical structures (ventral caudate) and the occipital cortex. Several of these regions including the calcarine cortex were also reported by Skidmore et al. [27] to show decreased efficiency in PD patients compared to controls. Yet, it is difficult to directly compare these findings with the present results, as efficiency is an inverse measure of the shortest path length which does not speak to the number of connections for a particular node. The observed connectivity differences, in particular in the frontal and occipital cortex, might be related to non-motor symptoms typical for PD patients such as executive dysfunction, attention problems [65], [66], [67], [68] or lower performance in visuo-spatial tasks [69], [70], [71]. The results nicely dovetail and extend findings from an fMRI-study of Cardoso and colleagues [52]. They used a flickering checkerboard task and a facial perception paradigm to investigate the visual system in PD patients. They reported decreased activity in primary visual cortex bilaterally in PD patients as compared to healthy volunteers during the checkerboard task and increased activity in the fusiform gyrus in the facial perception task. The authors of that study concluded that PD patients show significant changes in the visual system even before visual symptoms become clinically evident.

Future studies might examine how network differences change depending on medication status and type of medication or relate fMRI connectivity findings to PET measurements of dopamine levels.

On the other hand, patients had a higher degree of connectivity in the pre-central cortex, superior parietal cortex, precuneus, cingulate gyrus, supplementary motor area and inferior temporal regions. Higher connectivity in PD patients in cortical areas of the motor network might reflect compensatory mechanisms, which is indicated by many previous studies investigating motor control in PD patients [58], [59], [60], [61]. Mallol and colleagues [59], for instance, reported an increased participation of parietal-lateral premotor circuits in the execution of sequential motor tasks expressed by a hyperactivation in these areas compared to healthy controls.

### 5 Limitations and Conclusions

Our main motivation for further parcellating the AAL template ROIs [32] was the observation that these ROIs are very inhomogeneous which can be assessed from Figure 1. Using subdivisions of these ROIs led to increased homogeneity. At the same time we did not deviate strongly from the parcellation scheme which was used in many previous analyses on various neurological and psychiatric disorders [28], as each AAL ROI was sub-divided into only four sub-ROIs on average. Increasing the number of sub-ROIs improves the ROI homogeneity but on the other hand also increases the complexity of the data and the number of statistical tests to be performed when investigating between group effects. The parcellation of the brain into 343 regions was thus a compromise. The larger ROI homogeneity revealed the resting-state networks which were not observed with the coarser parcellation (Figure 1 and 4). Still, the number of ROIs was feasible when calculating complex network properties, displaying the network or performing corrections for multiple testing. The fMRI data of the present study was acquired with a gap of 2 mm which is certainly not optimal for deriving a brain parcellation. Although a somewhat lower ROI homogeneity can be expected with finer spatial resolution, we do not expect any bias being introduced for our statistical analyses and between-group comparisons.

Unfortunately, no data on cognitive or affective functions were available in the current set of PD patients. In light of the first papers that explored how specific graph-theory based network measures relate to cognitive or affective processes [72], [73], [74] this seems a fruitful avenue for further research. Our data does not allow us to study the role of medication and we are not able to rule out that some of the observed effects are medication related. Future work should investigate how network changes depend on disease stage and/or medication type (for instance, L-Dopa vs. DA agonists).

To summarize, we provide evidence for altered resting-state networks on a global, intermediate and local level in PD patients. Whereas previous studies on functional connectivity in PD focused on specific resting-state networks [10], a few network nodes [11] or only single connectivity measures such as nodal and global efficiency [27] here we explored whole-brain intrinsic connectivity employing various graph-theory based measures in a large patient sample. The results demonstrate the usefulness of our methodological approach to bring out local and global group differences with satisfying statistical and spatial sensitivity. We are convinced that the ROI and voxel level analyses complement each other as changes in functional connectivity on a smaller scale than the ROI-size are only apparent in the voxel level analysis but not on ROI-level. The voxel level analysis also might be used to help to study potential biases introduced by the choice of ROIs. The network parameters used in the present study might be interesting biomarkers to track disease state and characterize subtypes of PD patients related to cognitive dysfunctions or other non-motor symptoms.

## Supporting Information

Figure S1 Representation of the brain parcellation conducted in the present analysis. A) Parcellation of the whole brain into 343 ROIs. B) Parcellation of the right caudate.
(TIF)

Figure S2 The z-degree in the cuneus, calcarine and posterior cingulate cortex as a function of the sparsity for ROIs where we observed significant group effects comparing controls (blue circles) and PD patients (red squares). Critically, the reported effect does not depend on the sparsity.
(TIF)

Figure S3 Between group effects in degree centrality. Voxel-level (cluster defining threshold p = 0.005; cluster size threshold k>65; cluster wise significance p = 0.05 uncorrected) and ROI-level data (blue areas; considering only nodes in modules 4 and 6) are presented. A) Regions with a larger degree in healthy controls compared to patients. B) Regions with a larger degree for PD patients compared to controls.
(TIF)

Table S1 Brain regions showing a high degree centrality (control group; t>6.0). MNI-coordinates, t-scores and anatomical regions of local maxima.
(DOCX)

Table S2 Nodes in the visual (module 4) and sensorimotor network (module 6) showing the strongest between group effects in degree centrality.
(DOCX)

Table S3 Group differences in degree centrality (voxel degree analysis; uncorrected).
(DOCX)
