# Supplementary figures and images for: Altered Resting State Brain Networks in Parkinson’s Disease
Source: PLoS One. 2013 Oct 28;8(10):e77336. doi: 10.1371/journal.pone.0077336 (PMC3810472; doi:10.1371/journal.pone.0077336)

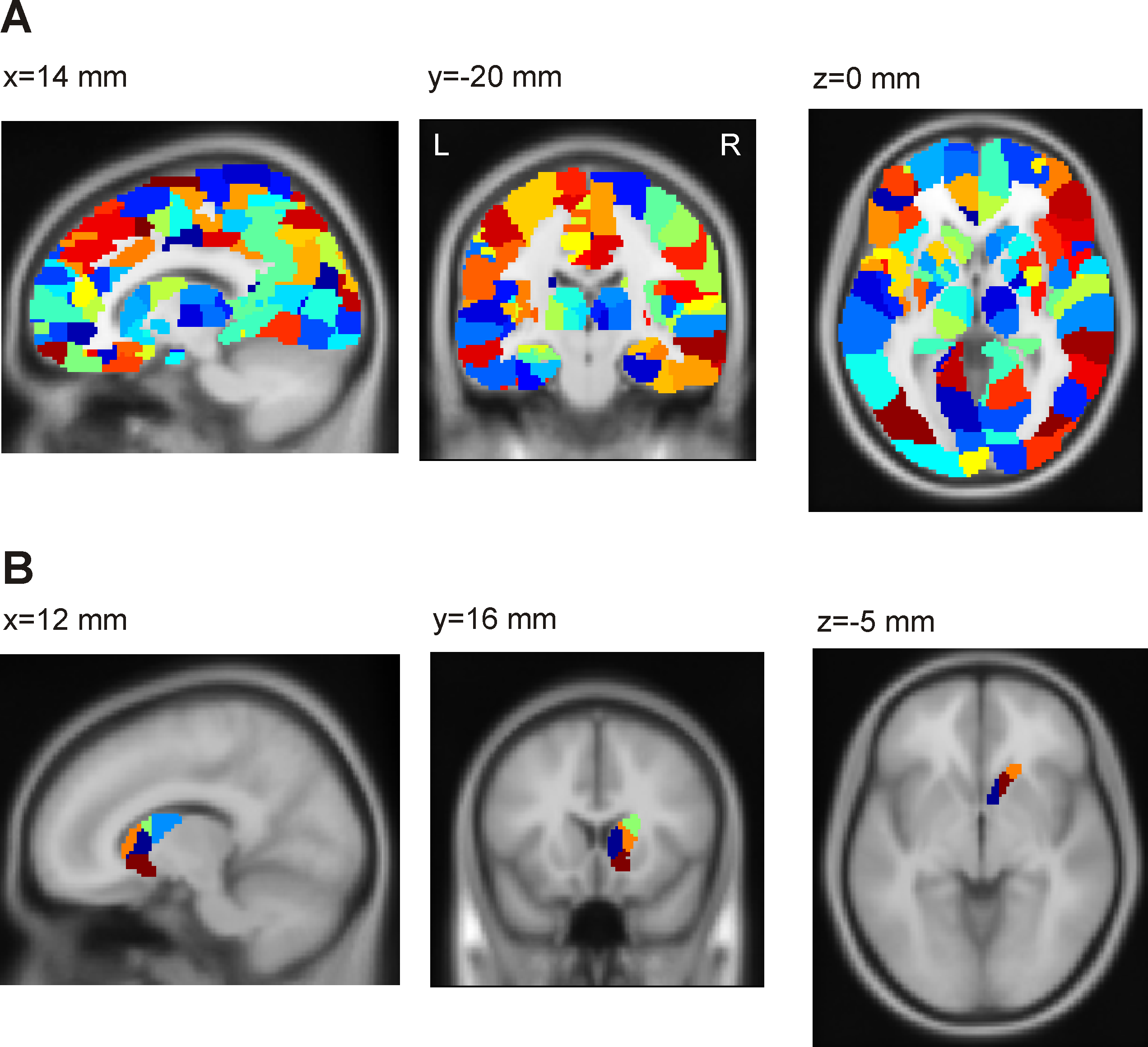

Supplement: Figure S1 — Representation of the brain parcellation conducted in the present analysis. A) Parcellation of the whole brain into 343 ROIs. B) Parcellation of the right caudate. (TIF) [file pone.0077336.s001.tif]

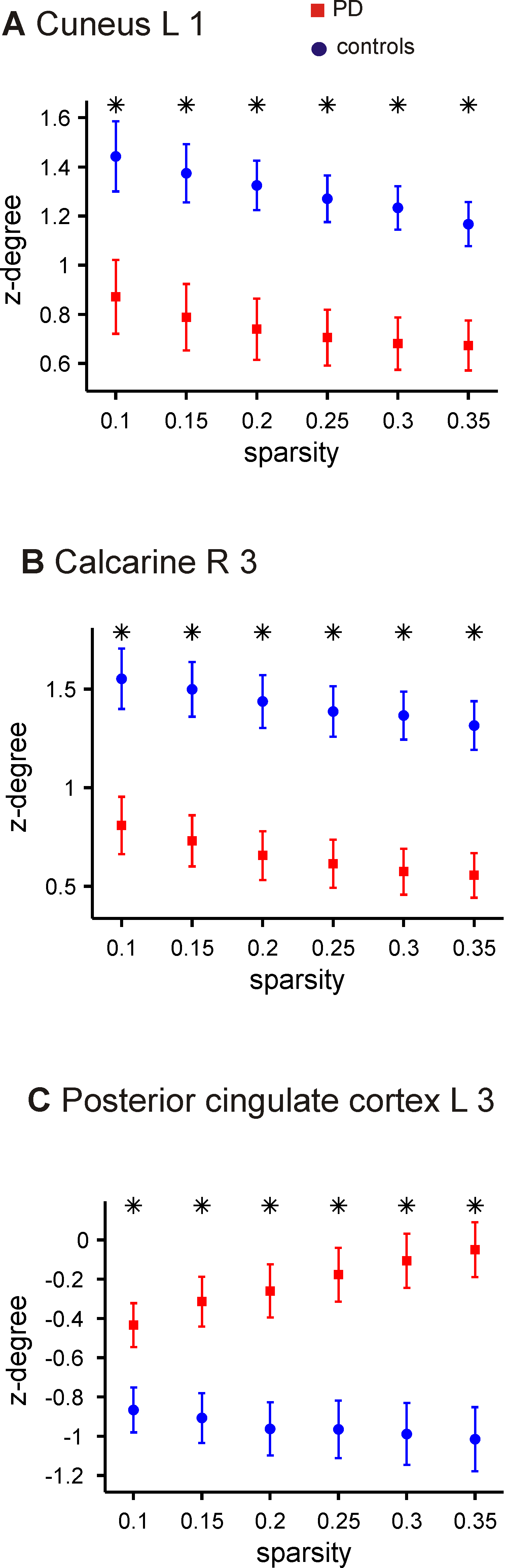

Supplement: Figure S2 — The z-degree in the cuneus, calcarine and posterior cingulate cortex as a function of the sparsity for ROIs where we observed significant group effects comparing controls (blue circles) and PD patients (red squares). Critically, the reported effect does not depend on the sparsity. (TIF) [file pone.0077336.s002.tif]

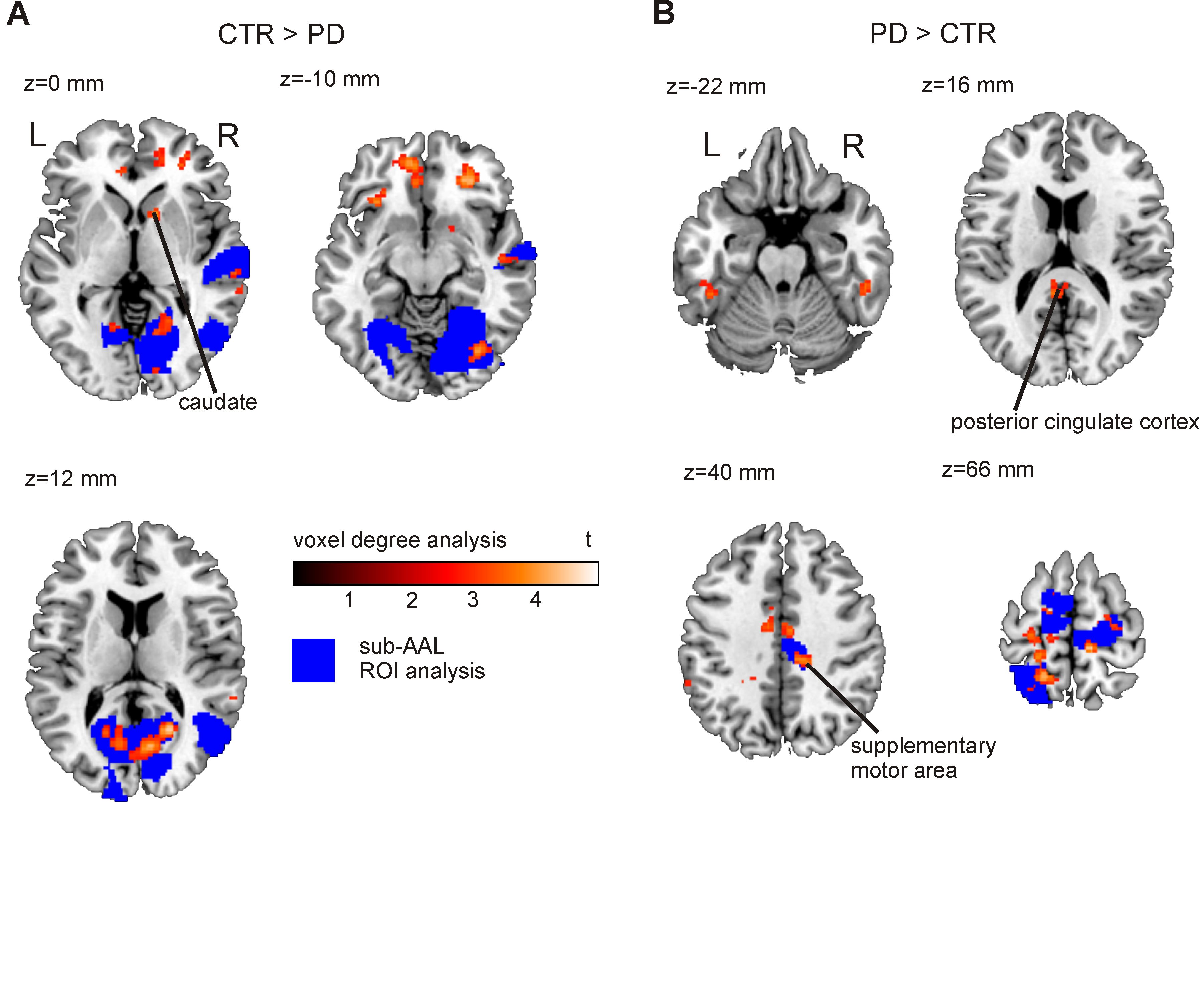

Supplement: Figure S3 — Between group effects in degree centrality. Voxel-level (cluster defining threshold p = 0.005; cluster size threshold k>65; cluster wise significance p = 0.05 uncorrected) and ROI-level data (blue areas; considering only nodes in modules 4 and 6) are presented. A) Regions with a larger degree in healthy controls compared to patients. B) Regions with a larger degree for PD patients compared to controls. (TIF) [file pone.0077336.s003.tif]
