# Supplementary material for: Altered Resting State Brain Networks in Parkinson’s Disease
Source: PLoS One. 2013 Oct 28;8(10):e77336. doi: 10.1371/journal.pone.0077336 (PMC3810472; doi:10.1371/journal.pone.0077336)
Supplement: Table S1 — Brain regions showing a high degree centrality (control group; t>6.0). MNI-coordinates, t-scores and anatomical regions of local maxima. (DOCX) [file pone.0077336.s004.docx]

**Table S1.** Brain regions showing a high degree centrality (control group; t>6.0). MNI-coordinates, t-scores and anatomical regions of local maxima.

local maxima t-score Anatomical region

(x y z) [mm]

-16 -78 36 18.60 Cuneus L

-12 -66 10 18.59 Calcarine L

2 -72 22 18.40 Cuneus R

-46 20 -16 12.73 Temporal Pole Sup L

-54 18 -6 10.85 Inf Frontal Gyrus L

-60 8 -4 8.33 Temporal Pole Sup L

-36 -4 58 11.87 Precentral L

66 -2 2 10.44 Temporal Sup R

58 -16 10 9.07 Temporal Sup R

60 10 -2 8.91 Temporal Pole Sup R

50 20 -10 10.10 Frontal Inf Orb R

-66 -20 -6 9.80 Temporal Mid L

-54 -34 14 9.17 Temporal Sup L

-26 -8 64 9.16 Frontal Sup L

-46 -72 10 9.07 Occipital Mid L

-42 -76 16 7.76 Occipital Mid L

46 -20 6 8.14 Heschl R

36 -20 2 6.51 Insula R

64 -30 -4 8.13 Temporal Mid R

38 0 56 7.88 Frontal Mid R

28 -8 64 7.52 Frontal Sup R

-32 26 -8 7.70 Frontal Inf Orb L

-62 -16 8 7.57 Temporal Sup L

-60 -24 10 5.65 Temporal Sup L

56 -12 -16 7.40 Temporal Mid R

46 6 48 7.39 Precentral R

50 -32 16 7.27 Temporal Sup R

-36 -82 4 7.00 Occipital Mid L

-62 -48 14 6.90 Temporal Sup L

46 -10 54 6.68 Pre-central R

0 46 32 6.48 Frontal Sup Medial L

-8 -14 10 6.13 Thalamus L

-10 -28 -2 6.13 Thalamus L

-28 -34 64 6.00 Postcentral L
