# Supplementary material for: Altered Resting State Brain Networks in Parkinson’s Disease
Source: PLoS One. 2013 Oct 28;8(10):e77336. doi: 10.1371/journal.pone.0077336 (PMC3810472; doi:10.1371/journal.pone.0077336)
Supplement: Table S2 — Nodes in the visual (module 4) and sensorimotor network (module 6) showing the strongest between group effects in degree centrality. (DOCX) [file pone.0077336.s005.docx]

**Table S2.** Nodes in the visual (module 4) and sensorimotor network (module 6) showing the strongest between group effects in degree centrality.

Anatomical region local maxima mean degree p

(x y z) [mm] CTR PD

A) Module 4 (visual network)

Calcarine L 2 CalL2 -11 -61 11 1.0 0.5 0.016

Calcarine L 3 CalL3 -12 -72 10 0.9 0.4 0.017

Calcarine R 1 CalR1 13 -82 8 1.1 0.4 0.001

Calcarine R 3 CalR3 14 -62 12 1.4 0.6 0.0001

Cuneus L 1 CunL1 -7 -76 29 1.3 0.7 0.0002

Cuneus R 1 CunR1 14 -72 29 1.2 0.7 0.002

Fusiform L 1 FusL1 -32 -68 -14 1.2 0.8 0.015

Fusiform R 2 FusR2 31 -48 -14 0.5 0.1 0.047

Fusiform R 3 FusR3 32 -66 -13 1.3 0.7 0.004

Lingual L 2 LinL2 -16 -64 -4 0.8 0.4 0.023

Lingual R 3 LinR3 15 -77 -5 1.1 0.6 0.003

Lingual R 4 LinR4 18 -59 -4 0.9 0.3 0.005

Occipital Inf R 2 OIR2 41 -76 -7 0.7 0.2 0.026

Occipital Sup L 4 OSL4 -13 -95 13 0.3 -0.2 0.034

Occipital Sup R 3 OSR3 27 -74 41 1.0 0.7 0.027

Temporal Mid R 2 TMR2 50 -64 11 1.5 0.9 0.001

Temporal Sup R 3 TSR3 60 -19 -1 0.9 0.4 0.007

B) Module 6 (related to the sensorimotor network)

Cingulate Cortex Mid R 1 CCMR1 9 -25 41 0.3 0.7 0.048

Paracentral Lobule L 1 PLL1 -12 -19 66 0.0 0.5 0.019

Paracentral Lobule L 3 PLL3 -8 -21 75 0.1 0.6 0.036

Paracentral Lobule R 1 PLR1 8 -29 64 -0.2 0.3 0.048

Parietal Sup L 1 PSL1 -25 -53 65 0.1 0.8 0.002

Postcentral R 1 PosR1 17 -40 78 -0.8 -0.2 0.016

Precentral R 3 PreR3 22 -23 70 0.2 0.7 0.039

Supp Motor Area L 3 SMAL3 -7 -5 70 0.2 0.8 0.027

**Notes:** Network nodes in modules 4 (A) and 6 (B) showing the strongest between group effects (one-tailed permutation test α=0.05). The anatomical region according to the AAL atlas, the center of mass MNI coordinates of the ROIs, the node degree for controls (CTR) and patients (PD) and the p-values are listed.
