# Supplementary material for: Altered Resting State Brain Networks in Parkinson’s Disease
Source: PLoS One. 2013 Oct 28;8(10):e77336. doi: 10.1371/journal.pone.0077336 (PMC3810472; doi:10.1371/journal.pone.0077336)
Supplement: Table S3 — Group differences in degree centrality (voxel degree analysis; uncorrected). (DOCX) [file pone.0077336.s006.docx]

**Table S3.** Group differences in degree centrality (voxel degree analysis; uncorrected).

Anatomical region p (unc.) k local maxima T (cluster) (x y z) [mm] (peak)

A) CTR > PD

Temporal Pole Mid L 0.004 162 -38 10 -32 5.09

Temporal Mid R 0.017 102 54 -12 -18 4.91

Calcarine R 0.000 1015 22 -60 12 4.63

Temporal Pole Sup R 0.027 85 42 14 -30 4.38

Frontal Inf Orb R 0.000 321 30 38 -10 4.35

Frontal Med Orb L 0.000 293 -8 48 -10 4.19

Occipital Inf R 0.002 184 34 -72 -8 4.14

Frontal Inf Orb L 0.032 79 -32 26 -8 4.02

Hippocampus L 0.021 94 -20 -4 -25 3.87

Caudate R 0.049 65 14 10 0 3.85

Frontal Sup Medial L 0.044 69 -14 42 22 3.81

Temporal Mid R 0.014 111 66 -38 4 3.81

B) PD > CTR

Parietal Sup L 0.000 738 -18 -52 62 4.94

SupraMarginal L 0.001 212 -66 -22 34 4.93

Postcentral R 0.022 93 12 -34 68 4.78

Cingulate Cortex Post L 0.049 65 -6 -44 14 4.42

Cingulate Cortex Mid R 0.031 81 12 -28 38 4.38

Temporal Inf L 0.038 74 -44 -44 -26 4.33

Precentral R 0.005 155 30 -24 60 4.20

Supp Motor Area L 0.000 346 -12 -8 52 4.18

Temporal Inf R 0.039 73 52 -38 -20 3.75

Cingulate Cortex Mid R 0.045 68 6 -10 40 3.66

Precentral L 0.030 82 -16 -12 68 3.46

Precentral R 0.037 75 14 -18 72 3.44

**Notes:** Clusters where differences in the voxel degree between patients and controls are observed (cluster defining threshold p<0.005; cluster size k>65; cluster wise significance α=0.05 uncorrected). Anatomical region, cluster level probability (uncorrected), number of voxels per cluster (k), local maxima in MNI coordinates and peak T-scores are listed.
